# The Family Homœopathist

**Published:** 1890-11

**Authors:** 


THE FAMILY HOMŒOPATHIST; OR, PLAIN DIRECTIONS FOR THE TREATMENT OF DISEASE. By E. B. Shuldam, M. D., Seventh edition; London, E. Gould & Son, 59 Moorgate Street.

This is a little 16mo volume of 150 pages, containing concise and plain directions for treatment of common diseases. The indications for the remedies are very clear and simple.

There is an introduction which, in a very few pages, is a very good defense of Homœopathy. This is followed by a chapter containing "hints on health," a chapter of "directions for taking the medicines," and a short Materia Medica. Then follow the different diseases with indications for the remedies as above mentioned.

Here and there we regret to say alternated remedies are recommended, marring the value of the book. Barring this defect, it is a clever and handy little volume for the laity. W. M. J.

